# Biochemical and morphological changes in endothelial cells in response to hypoxic interstitial edema

**DOI:** 10.1186/1465-9921-7-7

**Published:** 2006-01-13

**Authors:** Laura Botto, Egidio Beretta, Rossella Daffara, Giuseppe Miserocchi, Paola Palestini

**Affiliations:** 1 Department of Experimental, Environmental Medicine and Biotechnologies (DIMESAB), University of Milano-Bicocca, Via Cadore 48 20052 Monza, Italy

## Abstract

**Background:**

A correlation between interstial pulmonary matrix disorganization and lung cellular response was recently documented in cardiogenic interstitial edema as changes in the signal-cellular transduction platforms (lipid microdomains: caveoale and lipid rafts). These findings led to hypothesize a specific "sensing" function by lung cells resulting from a perturbation in cell-matrix interaction. We reason that the cell-matrix interaction may differ between the cardiogenic and the hypoxic type of lung edema due to the observed difference in the sequential degradation of matrix proteoglycans (PGs) family. In cardiogenic edema a major fragmentation of high molecular weight PGs of the interfibrillar matrix was found, while in hypoxia the fragmentation process mostly involved the PGs of the basement membrane controlling microvascular permeability. Based on these considerations, we aim to describe potential differences in the lung cellular response to the two types of edema.

**Methods:**

We analysed the composition of plasma membrane and of lipid microdomains in lung tissue samples from anesthetized rabbits exposed to mild hypoxia (12 % O_2 _for 3–5 h) causing interstitial lung edema. Lipid analysis was performed by chromatographic techniques, while protein analysis by electrophoresis and Western blotting. Lipid peroxidation was assessed on total plasma membranes by a colorimetric assay (Bioxytech LPO-586, OxisResearch). Plasma membrane fluidity was also assessed by fluorescence. Lipid microdomains were isolated by discontinuous sucrose gradient. We also performed a morphometric analysis on lung cell shape on TEM images from lung tissue specimen.

**Results:**

After hypoxia, phospholipids content in plasma membranes remained unchanged while the cholesterol/phospholipids ratio increased significantly by about 9% causing a decrease in membrane fluidity. No significant increase in lipid peroxidation was detected. Analysis of lipid microdomains showed a decrease of caveolin-1 and AQP1 (markers of caveolae), and an increase in CD55 (marker of lipid rafts). Morphometry showed a significant decrease in endothelial cell volume, a marked increase in the cell surface/volume ratio and a decrease in caveolar density; epithelial cells did not show morphological changes.

**Conclusion:**

The biochemical, signaling and morphological changes observed in lung endothelial cell exposed to hypoxia are opposite to those previously described in cardiogenic edema, suggesting a differential cellular response to either type of edema.

## Background

The interstitial compartment of the lung is kept at a subatmospheric pressure in physiological conditions, a feature shared by other compartments where extravascular water is kept at a least amount. In the lung, a relatively "dry" interstitial space allows a minimum thickness of the air-blood barrier to optimize gas diffusion. A rise in extravascular lung water may occur because of an increase in the pressure gradient across the microvascular barrier and/or by an increase in perm-porosity of the endothelial barrier. The first case, the so called cardiogenic lung edema, may represent the consequence of left ventricular failure with increased left atrial and pulmonary capillary pressure. Conversely, hypoxia exposure may fall into the second case as it may augment microvascular permeability. Severe lung edema is indeed a life threatening complication of high altitude exposure with presence of protein rich fluid in the alveolar spaces.

An important finding concerning the initial phase of edema development in both models is that a minor increase in extravascular water, about 5%, leads to a marked increase in interstitial pressure (from about -10 to about 5 cm H_2_O [1]), indicating a fairly low compliance of the lung extracellular matrix that obviously represents a strong "tissue safety factor" against edema development as it balances further microvascular filtration [2]. It was also found that in interstitial lung edema, some degree of disorganization of the extracellular matrix occurs, despite its strong mechanical resistance, particularly at the expense of proteoglycans (PGs) [3]. These molecules are responsible for the structural integrity of pulmonary interstitium as they control fluid dynamics through their influence on microvascular permeability and tissue compliance. Furthermore, proteoglycans are also involved in cell-cell and cell-matrix interactions and in the cytokine network [4] as they regulate the traffic of the molecules within the interstitial space and promote interactions. A possible correlation between matrix disorganization and cellular function was documented in the cardiogenic model of interstitial edema as changes in composition of plasma membrane lipid microdomains involved in signal-transduction [5]. These findings led to hypothesize a specific "sensing" function by lung cells resulting from a perturbation in cell-matrix interaction [6]. We may reason that the cell-matrix interaction may differ between the two types of edema as a difference was found in the sequential degradation of PGs family and in the interaction properties of PGs to some matrix components [7,8]. Indeed, in the cardiogenic model we found a major fragmentation of high molecular weight chondroitin sulphate PGs of the interfibrillar matrix, while in hypoxia the fragmentation process mostly involved the intermediate molecular weight heparansulphate PGs, such as perlecan of the basement membrane. Furthermore, for a similar increase in extravascular water, PGs degradation, as judged from total hexuronate recovery, was greater in hypoxia [7]. Based on these considerations, we aim to describe potential differences in the lung cellular response to the two types of edema that imply differences in the process of disorganization of the extracellular matrix. We performed a biochemical and morphometric study focusing in particular on the plasma membrane bilayer lipid pattern, including a particular subset of phospholipids (lyso-phospholipids and plasmalogens) that are implicated in the oxidant- antioxidant phenomena and on lipid microdomains (caveolae and lipid rafts).

## Methods

### Chemical

The reagents used (analytical grade) and HPTLC plates (Kieselgel 60) were purchased from Merck GmbH (Darmstadt, Germany). CAPS, MES, Percoll, PMSF, HRP-CTB were from Sigma Chem. Co. (Milano, Italy). Antibody against caveolin-1 (C2297) and flotillin (F65020) were from Transduction Labs (Lexington, KY, USA). Antibody against aquaporin-1 (sc-9878) was from Santa Cruz Biotechnology (CA, USA). CD55 (1A10) was from BD Pharmigen. Antibody against actin (A 2066) was from SIGMA. All the material for the electrophoresis was from BioRad, (Milano, Italy). Autoradiographic films was from Amersham Pharmacia Biotech (Uppsala, Sweden).

### Lung tissue preparation and plasma membrane purification

General preparation. Experiments were done in rabbits (2.5 ± 0.5 (SD) Kg body wt) anesthetized with a mixture of 2.5 ml/kg of 50% urethane (wt/vol, in saline solution) and 40 ml/kg body wt of ketamine injected into an ear vein. Subsequent doses of anesthetic were administered during the experiments judging from the arousal of ocular reflexes.

The study was based a protocol accepted by D.L. 116/1992, art.3, 4, 5 and performed according to the established rules of animal care.

The trachea was cannulated. We considered the following groups of animals for biochemical determinations:1) animals exposed to room air breathing sacrificed immediately after anesthesia and tracheotomy (control N = 5); 2) animals exposed to room air and left to breath in anesthesia for up to 3 h (sham N = 4); 3) animals exposed to hypoxia (12 % O_2 _in nitrogen) for 3 h (N = 4); 4) animals exposed to hypoxia for 5 hours (N = 3).

We perfused the lungs for about 5 min at room temperature with mammalian Ringer's solution (without calcium) containing nitroprusside (20 mg/ml). Nitroprusside is a donor of nitric oxide; however this effect should be present both in control and treated animal samples; therefore the observed differences in membrane protein response when comparing control and sham to treated animals should be due to the specific conditions caused by hypoxia. After this, the lungs were flushed with 50 ml of solution 1 (0.25 M sucrose, 20 mM Tricine pH 7.4 and 40 μg/ml of the protease inhibitors aprotinin, chymostatin, leupeptin and antipapain), excised from the chest and immersed in ice cold solution 1.

We also estimated the level of lipid peroxidation in control, sham, hypoxia exposure and saline induced lung edema (i.v. infusion 0.5 ml/kg min for 3 h) that mimics cardiogenic edema: in these animals, we added butylate hydroxytoluene in solution 1 to reach a concentration 0.2 mM.

The lung tissue was finely minced at 4°C and homogenated in solution 1, then filtered sequentially through 53 and 30 μm filters. The homogenate was subjected to centrifugation (1,000 g for 10 min) at 4°C, and the supernatants were saved. The resulting pellet was resuspended in 3 ml of buffer and subjected again to centrifugation as above. The pooled supernatants were overlaid over 25 ml of 30% Percoll in buffer. After centrifugation using a SW28 rotor at 84,000 g for 45 min at 4°C, we collected a single membranous band (about 1 ml) readily visible at about 2/3 from bottom of the tube. To reduce the volumes and concentrate the membranes, the bands were pelleted by first diluting the suspension 3 fold with PBS before centrifugation at 100,000 g for 20 min at 4°C. These membrane fractions were collected and called PMC (for control), PMH3 and PMH5 (for 3 and 5 hours of hypoxia) respectively, and aliquots were taken for different analysis.

### Isolation of detergent-resistant fraction

The plasma membrane pellets (PMC and PMH3) were resuspended in 1 ml of MBS buffer (25 mM of MES buffer, pH 6.5, containing 150 mM NaCl, 1 mM phenylmethylsulfonylfluoride and 75 units/ml aprotinin) and we determined its protein content (BCA methods). Next, we took a volume containing 4.5 mg of protein, a quantity required for each gradient procedure. In order to maintain a constant protein/detergent ratio in all experiments, we added MBS buffer containing Triton X-100 up to a volume of 2 ml to reach a final Triton concentration of 1%. All the procedure was carried on ice for 20 min to maintain the integrity of lipid rafts. Finally, the 2 ml were diluted with an equal volume of 80% (w\v) sucrose in MBS lacking Triton X-100 and placed at the bottom of a tube where a discontinuous sucrose concentration gradient was created (40, 30, 5 % sucrose, from bottom up) in MBS lacking Triton X-100. After centrifugation at 250,000 g for 18 hrs at 4°C with a TW-41 rotor (Beckman Instruments), 1 ml fractions were collected from the top of the gradient and submitted to further analysis. From now on, fraction #5 (from the top) is referred as DRF (Detergent Resistant Fraction); fractions from # 6 to 8 as IDF (Intermediate Density Fraction); fractions from # 9 to 12 as HDF (High Density Fraction).

### Phosphorus analysis and fluorescence spectroscopy

Aliquots of PMC, and PMH3 and PMH5 from all animals were used for phospholipid phosphorus determination [9]. Data were expressed as micromoles per milligrams of protein. The membrane fluidity of different samples was assessed by fluorescence anisotropy measurements of the fluorescent probe 1, 6-diphenyl-1, 2, 5-hexatriene (DPH) as described [10] with minor modification. A suspension of PMC, PMH3 and PMH5, containing ~200 nmol of phosphorus per 1.5 ml of PBS was used. The fluorescent probe molecule DPH was added to membrane suspension at a final concentration of 10^-3 ^M. Light scattering was corrected by using a blank containing the sample but not DPH. Membranes with and without fluorescent probe were incubated in the dark under stirring for 45 min at 37°C and were used for fluorescence polarization studies immediately after preparation. A polarization spectrofluorimeter (Cary Eclipse, Varian) with fixed excitation and emission polarization filters was used to measure fluorescence intensity parallel (I_pa_) and perpendicular (I_per_) to the polarization plane of the exciting light [10]. Excitation and emission wavelengths were 360 and 430 nm, respectively. Fluorescence anisotropy was calculated as *r *(I_pa_-I_per_/I_pa_+I_per_). The sample was continuously stirred with a microstirrer, and the temperature (37°C) was monitored by a thermistor in the cuvette.

### Lipids and fatty acid analysis

Aliquots of PMC, PMH3 and PMH5, were submitted to lipid extraction [10]. An organic phase (containing all lipids with the exception of gangliosides) and an aqueous phase (containing gangliosides) were obtained. The lipids were separated on HPTLC plates. The phospholipids from PMC, PMH3 and PMH5 were chromatographed in *solution B *(chloroform:methanol:acetic acid:water, 60:45:4:2, each by vol). The cholesterol, from plasma membranes, was chromatographed in *solution D *(hexane:diethylether:acetic acid, 20:35:1, each by vol). In the case of neutral glycosphingolipids (GLS), the lipids extracted were submitted to alkaline methanolysis (1 h at 37°C in 0.6 N NaOH in methanol) to remove contaminating phospholipids. After extensive dialysis, the GLS were chromatographed in *solution E *(chloroform:methanol:water, 110:40:6, each by vol). For the analysis of the plasmalogens, the phospholipids were chromatographed in *solution B*. The plates were then exposed to HCl vapors for 10 min and subsequently chromatographed in *solution F *for second dimension (chloroform:methanol:acetone:acetic acid:water, 50:15:15:10:5, each by vol).

For the analysis of the lysophospholipids, the phospholipids were chromatographed in *solution B *and subsequently chromatographed in *solution G *for second dimension (chloroform:methanol:88% formic acid, 65:25:10, each by vol).

Phospholipids and cholesterol were visualized with anisaldehyde, and neutral glycosphyngolipids with orcinol. The plates were scanned with Bio-Rad system and spot identification, and quantification was accomplished by comparison with authentic standard lipids. Aliquots of different total lipids extracted, corresponding to 100–150 nmol of phosphorus, were submitted to fatty acid analysis [10].

The double bound index (DBI), commonly considered as an index of the ratio of saturated to unsaturated fatty acids, was calculated as follows: ∑ saturated fatty acids/∑ unsaturated fatty acids, where ∑ unsaturated f.a. is obtained by adding the percentage of each unsaturated fatty acid multiplied by the number of the double bounds in its molecule.

### Lipid peroxidation

Lipid peroxidation was assessed on total plasma membranes in 1 animal for each group (control, sham, 3 h of hypoxia exposure) by a colorimetric assay (Bioxytech LPO-586, OxisResearch) of malondialdehyde (MDA) as indicator of peroxidation. Data of MDA from lung tissue were expressed as nmol/μmol of plasma membrane phospholipidic phosphorous sampled.

### Protein analysis

Aliquots of PMC, PMH3 and PMH5 and all fractions collected from the gradient, were submitted to trichloroacetic acid precipitation. The pellets, washed with acetone, were suspended in water and protein quantity determined by BCA method (SIGMA, USA). Thereafter, 50 μg of PMC, PMH3 and PMH5 and 10 μg of proteins collected from the gradient, respectively, were loaded on SDS-PAGE; 10% -polyacrylamide gel, and submitted to electrophoresis. Subsequently, the proteins were transferred to membranes that were stained with Ponceau S to assess protein loading by densitometry (BIORAD Densitometry 710, program Quantity one) [6,11]. We compared on our samples the densitometry of the whole lane for protein loading obtained from total plasma membranes and all gradient fractions from control and treated animals.

Actin contents was used to normalize total plasma membrane protein contents. This normalisation is not possible for proteins from fractions obtained from discontinuous sucrose concentration gradient because actin contents differs among these fractions [12].

Subsequently the membranes were submitted to Western blotting. After blocking, blots were incubated for 2 h with the primary antibody diluted in PBS-T/milk (anti-cav1 1:1000, anti-flotillin-1 1:250, anti AQP1 1:100, anti CD55 1:100, anti actin 1:1000). Then, blots were incubated for 2 hr with horseradish peroxidase-conjugated anti-mouse/goat IgG (5,000–10,000-fold diluted in PBS-T/milk). The protein samples were obtained from 3 controls and 3 treated animals. Proteins were detected by the SuperSignal detection kit (Pierce, Rockford, IL). We performed in parallel immunoblot analysis of samples from one control and one treated animal for total plasmamembrane, and proteins from all gradient fractions. Immunoblot bands were analyzed by BIORAD Densitometry 710.

### Statistical analysis

Biochemical determinations were repeated at least three times for each animal. Biochemical results were expressed as means ± SD, averaging data from the different animals. The significance of the differences among groups was determined using one-way ANOVA and *t*-test.

### Morphometry

The morphometric analysis was done in the following animal groups:1) rabbits exposed to room air breathing sacrificed immediately after anesthesia and tracheotomy (control; N = 2); 2) rabbits kept under anesthesia for 3 hours (sham 3 h; N = 3); 3) rabbits kept under anesthesia for 5 hours (sham 5 h; N = 2), 4) rabbits exposed to hypoxia (12% O_2 _in nitrogen) for 3 h (N = 3); 5) rabbits exposed to hypoxia for 5 hours (N = 3); 6) rabbits receiving i.v. saline infusion (0.5 ml/kg min for 3 h; N = 4) to cause an increase in lung extravascular water similar to that caused by hypoxia exposure.

For morphometric analysis we performed lung perfusion-fixation in situ following a technique carefully detailed in a previous paper [13]. Animals were killed by an overdose of anesthetic just prior to the perfusion procedure; next, with pleural sacs intact, we infused through the pulmonary artery first saline (11.06 g NaCl/l plus 3% dextran T-70 and 1,000 U heparin/dl, 350 m O sm) and then fixative (phosphate buffered 2.5% glutaraldehyde plus 3% dextran T-70, 500, under a pressure head of 15 cm H_2_O.

Tissue samples were obtained following a stratified random sampling procedure from ventral (top) to dorsal (bottom) lung region and immersed in 2.5% glutaraldehyde for 1 hour at room temperature and subsequently processed for resin embedding.

For light microscopy analysis, 1 μm thick sections were obtained and stained with methylene blue. For electron microscopy, 60 nm thick sections were obtained; they were mounted on uncoated 200-mesh copper grids, stained with uranyl acetate and lead citrate, finally observed in a Zeiss EM900 electron microscope.

### Morphometry at light microscopy

Micrographs were originally obtained at 100× (Olympus BX51) and brought to a final magnification of 2600× on the computer video screen for morphometric measurements that were done according to established stereological techniques [14]. We evaluated the surface area of the capillaries (Sc) from the number of intersections of test lines with the boundary profile of the capillaries according to Sc = (2 × I)/L_t_, where L_t _is the total length of all the test lines of the grid (length of each test line = 8.57 μm). The data base for morphometric analysis at light microscopy was obtained from about 1000 fields from each animal group.

### Morphometry at transmission electron microscopy of the thin portion of the air-blood barrier

The thin portion of the air-blood barrier is primarily involved in gas diffusion and corresponds to septal regions were only a fused basement membrane separates endothelium and epithelium. In these regions we performed a morphometric evaluation of endothelial, epithelial and interstitial compartments on micrographs obtained at 22,000×, brought at a final magnification of 66,000×.

The mean arithmetic thickness (τ) of the interstitial layer separating the endothelial and the epithelial compartments was determined using a multipurpose M168 grid (40) as given by: τ = (d·P)/[2·(I tot)], were d is the length of test line (d = 0.174 μm), P being the number of points falling in the compartment and I tot being its overall surface boundary profile.

Volume densities (Vv) of endothelial and epithelial compartments were obtained by the point counting method, while total surface areas (Stot) of each of these compartments were obtained by the intersection counting method, using a cycloidal test system [15]. For a given compartment, total surface area and volume density are linked by the relationship Stot = Vv × Sv, where Sv is defined as surface density, namely surface area per unit volume (μm^2^/μm^3^). Surface density is given by Sv = 2 × I_i_, where I_i _is the number of intersections between the surface area and the test lines per unit length of test line (0.1855 μm).

We also evaluated the numerical density (N_v_) of plasmalemmal vesicles (PVs) in endothelial and epithelial cells; vesicles were identified by their morphology as being non-coated and 50–90 nm in diameter. Numerical density was obtained as N_v _= number of PVs/unit volume multiplied by a correction factor given by ( + T - 2*h*) where:  is the true mean diameter of the PVs (considered to average 70 nm, as commonly accepted in literature); T is the thickness of the ultrathin sections (60 nm); *h *is the depth by which a vesicle must penetrate the section before it is detected [14,16].

The data base for the analysis came, for each animal group, from about 150 counting fields randomly chosen on the micrographs.

For morphometric analysis, primary data (point, line intersection and vesicle counts) were summed over all the micrographs derived from each section and the parameters were computed as the ratio of sums. The parameters were then averaged over the various section samples. Data were expressed as means ± SE. The significance of the differences among groups was determined using one-way ANOVA and *t*-test.

An estimate of the extravascular water accumulation was obtained for the ratio between the weight of the fresh tissue samples and after drying in the oven at 70°C for at least 24 h (W/D ratio).

## Results

### Lipid analysis

The amount of phospholipidic phosphorus in plasma membranes, normalized to total protein quantity did not change significantly in hypoxic lungs relative to control (Table 1). Data referring to sham animals were pooled with control as they did not differ significantly.

**Table 1:** Lipid content of plasma membrane fractions in control (PMC; N of animals = 3) and after 3 (PMH3; N of animals = 3) and 5 hours of hypoxia exposure (PMH5; N of animals = 3).

	PMC	PMH3	PMH5
Phosphorus Phospholipid (μmol/mg protein)	1.10 ± 0.2 (8)	1.24 ± 0.25 (10)	1.2 ± 0.3 (7)
Cholesterol (nmol/mg protein)	247 ± 9.8 (10)	299 ± 23.7 (24) ^#^	296 ± 26.7 (17) ^#^
Cholesterol/Phospholipids (nmol/μmol)	0.224	0.241^#^	0.246^#^

The data are means ± SD; in parenthesis the number of determinations ; ^# ^*P *< 0.001 vs. control

Aliquots of samples were submitted to lipid extraction, and the different lipids (cholesterol, glycolipid, and phospholipid) were separated on HPTLC plates. The cholesterol concentration increased significantly at 3 h, remaining steady up to 5 h (P < 0.001) of hypoxia exposure and, consequently, also the cholesterol/phospholipids ratio increased significantly (Table 1). Some differences were found in the pattern of neutral glycolipids obtained from plasma membranes. The most abundantglycolipid, the lacto-*N*-*neo*tetraesosylceramide decreased in PMH3 and PMH5, relative to PMC (from 73 % to 65 %, respectively), whereas triesosylceramide increased from 8 % to 14 %, respectively, both changes being significant (P < 0.01).

**Figure 1:**
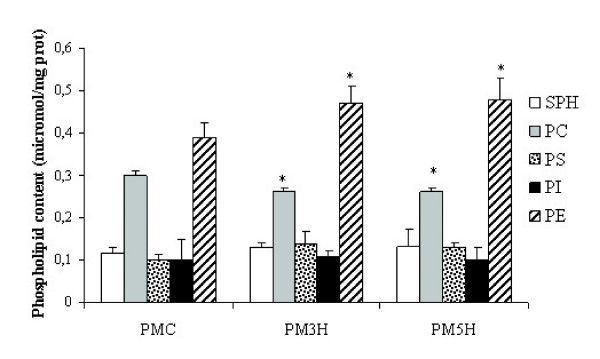
**Content of phospholipids in plasma membrane fractions. **Data were obtained from control (PMC) and treated lungs, after 3 h (PMH3) and 5 h (PMH5) of exposure to hypoxia and represent mean ± SD; data are from 3 animals in each condition (three determinations in each animal). SPH : sphingomyelin; PC : phosphatidylcholine; PS: phosphatidylserine; PI: phosphatidylinositol; PE: phosphatidylethanolamine. *P < 0.02 vs control.

The phospholipid pattern, normalized to protein quantity, is shown in Fig. 1. When comparing to control, only phosphatidylethanolamine (PE) and phosphatidylcholine (PC) showed significant differences. The PE increased by ~24 % in PMH3 and PMH5 (P < 0.02), while PC decreased by ~7% and ~13 % in PMH3 and PMH5, respectively. The PC/PE ratio increased from 1.3 in control, to 1.67 and 1.84 at 3 and 5 h of hypoxia, respectively. Phosphatidylglycerol quantity was similar in control and treated lungs, averaging ~3% of total phospholipids.

In PMC the amount of choline plasmalogen (included in PC) and ethanolamine plasmalogen (included in PE) were 0.024 and 0.16 nmoles/mg proteins, respectively. In PMH3, these values increased (0.048 and 0.215 nmoles/mg proteins, for choline and ethanolamine plasmalogen, respectively) while in PMH5, they returned towards control values (0.021 and 0.192 nmoles/mg proteins, respectively).

Lysophsophatidylethanolamine, as determined by 2D-HPTLC, was unchanged after hypoxia exposure and averaged about 0.07 nmoles/mg protein. Lysophsophatidylcholine was undetectable in all conditions.

### Lipid peroxidation

MDA values (nmol/μmol phosphorous) were 5.7 ± 0.32 (control plus sham), 6.8 ± 2.66 (hypoxia 3 h) and 6.51 ± 0.32 (saline infusion); the increase observed in hypoxia and saline infusion (19 and 14%, respectively) were not significant.

### Fatty acid analysis and fluorescence spectroscopy

Table 2 reports the percentage composition of total lipid fatty acids obtained from plasma membranes. A significant decrease of palmitic acid (16:0) was observed both in PMH3 and PMH5. Arachidonic (20:4) and arachidic acid (20:0) increased significantly only in PMH3, while miristic acid (14:0) increased significantly only in PMH5. These modifications in fatty acid composition caused a decrease, though not significant, of the DBI (Table 3).

**Table 2:** Fatty acid composition of total lipids in plasma membrane fraction in control (PMC; N of animals = 3) and after 3 (PMH3; N of animals = 3) and 5 hours of hypoxia exposure (PMH5; N of animals = 3)

*Fatty acid*	PMC	PMH3	PMH5
14:0	1.59 ± 0.37	1.57 ± 0.38	2.48 ± 0.73 *
16:0	38.49 ± 0.58	33.36 ± 2.1*	35.2 ± 2.16 *
16:1	2.83 ± 0.22	2.38 ± 0.49	3.6 ± 0.53
18:0	14.07 ± 1.8	13.50 ± 1.4	16.1 ± 3.10
18:1	18.8 ± 0.9	18.30 ± 2.6	18.3 ± 0.92
18:2	13.36 ± 2.3	13.60 ± 1.6	13.3 ± 1.06
20:0	0.78 ± 0.25	2.0 ± 0.7 ^§^	1.09 ± 0.17
20:1	0.42 ± 0.01	0.68 ± 0.9	0.53 ± 0.44
20:4	9.16 ± 0.2	12.46 ± 2.35 ^§^	10.43 ± 1.97

The data are means ± SD (n = 6). § P < 0.05 vs control; * P < 0.001 vs control

**Table 3:** Double bound index (DBI) and fluorescence anisotropy (r) in plasma membrane fraction in control (PMC; N of animals = 3) and after 3 (PMH3; N of animals = 3) and 5 hours of hypoxia exposure (PMH5; N of animals = 3).

	PMC	PMH3	PMH5
DOUBLE BOUND INDEX (DBI)	0.643 ± 0.01	0.563 ± 0.03	0.605 ± 0.02
FLUORESCENCE ANISOTROPY (r)	0.250 ± 0.009	0.269 ± 0.007*	0.265 ± 0.006*

The data are means ± SD (n = 6). * P < 0.001 vs control

Using the fluorescent probe of the membrane fluidity DPH, a significant increase of the anisotropy parameter *r *was detected in PMH3 and PMH5, indicating a decrease in fluidity of the plasma membrane (Table 3).

### Protein analysis in DRF

**Figure 2:**
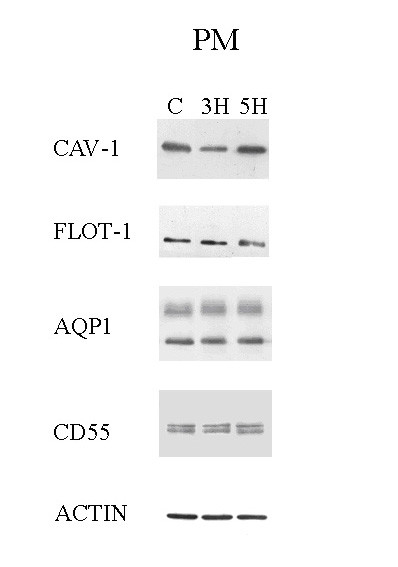
**Protein contents of total plasma membranes. **Caveolin-1, flotillin-1, AQP1, CD55 and actin contents in plasma membrane from tissue homogenates in control (C) and hypoxia (3 H, 5 H). At 3 H, caveolin-1 was significantly decreased.

**Figure 3:**
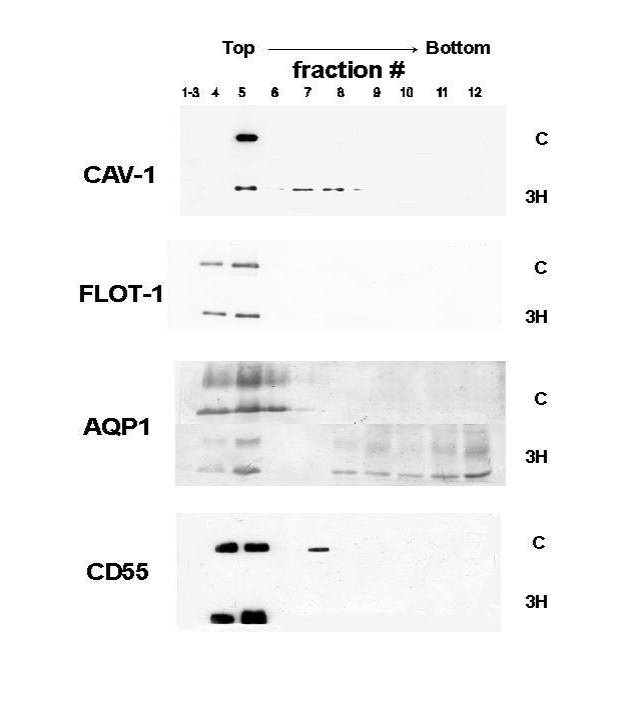
**Immunoblot analysis of plasma membrane proteins in sucrose gradient fractions. **Immunoblot analysis of Caveolin-1 (CAV-1), flotillin (FLOT-1), AQP1 and CD55 from control and after exposure to 3 h of hypoxia (C and 3 H, respectively). In hypoxia, CAV-1 and AQP1 decreased in fraction 5; FLOT-1 did not change while CD55 increased in fractions 4 and 5

The protein quantity of lipid microdomains obtained from DRF amounted to about 3% of total plasma membrane protein quantity and this value did not change significantly on comparing control to 3 h hypoxia exposure. Caveolin-1, flotillin-1, aquaporin-1 (AQP1), and CD55 were assessed by Western blotting analysis in total plasma membranes fractions (Fig. 2) and in sucrose gradient fractions (Fig. 3) of lung tissue samples from animals exposed to 3 h of hypoxia. Fig. 2 shows that the caveolin-1 content in total plasma membranes, evaluated from the densitometry, decreased significantly (P < 0.01) by about 36% at 3 h of hypoxia but returned towards control value at 5 h. AQP1, flotillin-1 and CD55 did not change in hypoxic lungs with respect to control as well as beta- actin.

Fig. 3 shows the protein distribution in the detergent resistant fractions after 3 h of hypoxia. Flotillin-1 content in fractions 4 and 5 was unchanged on comparing control to hypoxia. Caveolin-1 was enriched in fraction 5 in control, while it decreased about 7 fold in hypoxia in this fraction; furthermore it was also found in intermediate density fractions. AQP1 was mainly present in fractions 4–6 in control while in hypoxia it spread also towards higher density fractions. CD55 was mostly present in fractions 4 and 5 in control and in minor amount in fraction 7, while in hypoxia almost doubled in fractions 4 and 5.

### Lipid analysis in DRF

**Figure 4:**
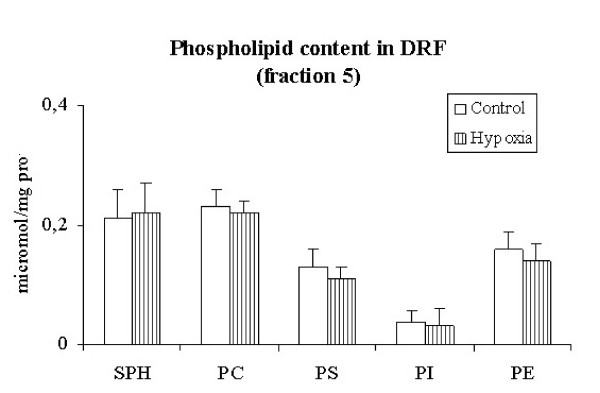
**Distribution of phospholipids in the plasma membrane detergent resistant fraction**. Distribution of different phospholipids in fraction 5 (detergent resistant fraction) in control and after exposure to 3 h of hypoxia (Control and hypoxia, respectively). SPH, sphingomyelin; PC, phosphatidylcholine; PS, phosphatidylserine; PI, phosphatidylinositol; PE, phosphatidylethanolamine

Cholesterol was enriched in DRF in control (878 ± 80, nmol/mg prot) and did not significantly change after 3 h of hypoxia (802 ± 77, nmol/mg prot). Fig. 4 shows that the phospholipid content in DRF, expressed as μ moles of phosphorous/mg protein, remained essentially unchanged after 3 h of hypoxia.

### Morphometry

The morphometric analysis did not show differences between sham and control, therefore the data were pooled. Table 4 shows that the average thickness τ of the interstitial space in the various groups of rabbits. Data relative to controls were pooled with those of sham referring to 3 and 5 h as no differences were found. As Table 4 shows, τ increased with hypoxia exposure, doubling significantly at 5 h; a similar increase occurred in the saline infusion group, indicating a similar degree of interstitial edema. Hypoxia also induced a remarkable increase in lung perfusion as indicated by the increase in surface area (Sc) of capillaries at 3 h, with a subsequent return towards control values at 5 h (Table 4).

**Table 4:** Thickness of the interstitial layer of the air-blood barrier (τ int, derived from transmission electron microscopy images) and surface density of pulmonary capillaries (Sc, from light microscopy images).

	τ int, μm	Sc cm^2^/cm^3^
CONTROL + SHAM	0.03 ± 0.004	803.15 ± 28.06
HYPOXIA 3 h	0.05 ± 0.002	1018.25* ± 11.27
HYPOXIA 5 h	0.06* ± 0.01	857.05 ± 19.72
CARDIOGENIC EDEMA	0.06^*$ ^± 0.01	890.84^$ ^± 47.47

Mean ± SD. * P < 0.05 vs control; $ data from ref. 13 for comparison

**Figure 5:**
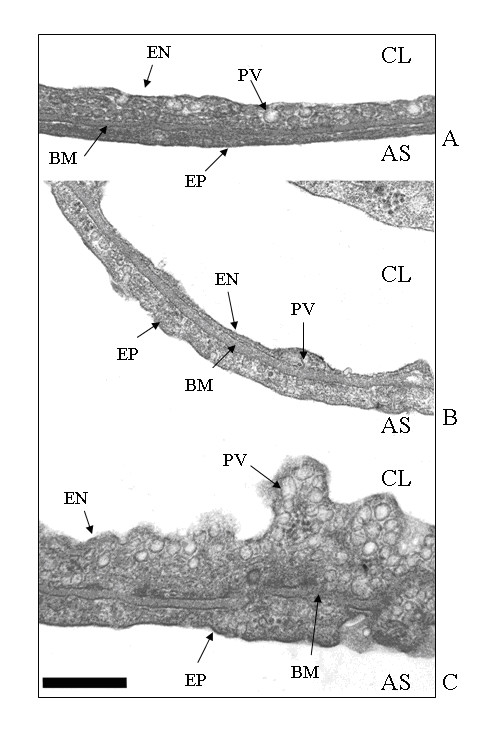
**Ultrastructural appearance of the thin portion of the air-blood barrier. **Micrographs at transmission electron microscope of the air-blood barrier in control lungs (A), in hypoxia (B) and in cardiogenic edema (C) at high magnification (x66000). CL, capillary lumen; AS, alveolar space; EN, endothelium; PV, plasmalemmal vesicle; BM, basement membrane; EP, epithelium. Scale bar = 0.5 μm.

Fig.5 shows high magnification (× 66000) micrographs of the thin portion of the air blood-barrier made of endothelial and epithelial cells, separated by a layer of fused basement membrane. Relative to control (A), hypoxia exposure (B) induced a thickening of the basement membrane, a considerable thinning of the endothelial layer but no appreciable changes in the epithelial layer. Fig. 5 C allows to evaluate the response of endothelial and epithelial cells of the air blood barrier in the cardiogenic edema group; for an increase in basement membrane thickness similar to that occurring in hypoxia, there was a considerable increase in cell volume and, particularly for endothelial cells, in surface area and in density of plasmalemmal vesicles.

**Figure 6:**
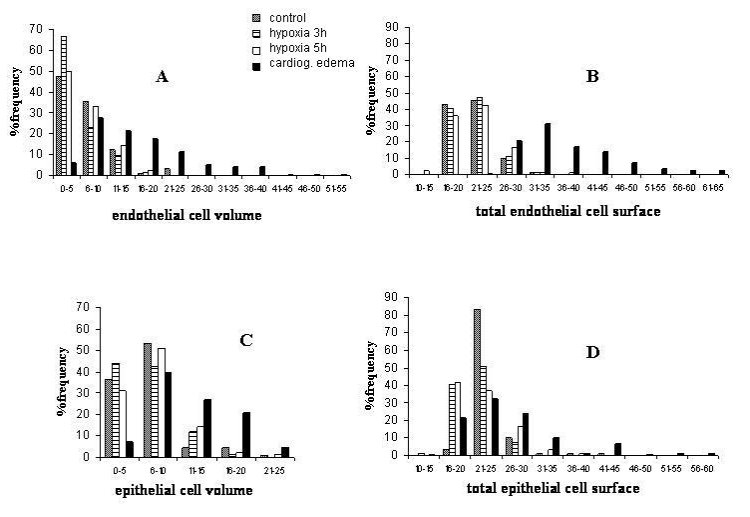
**Frequency distribution of volume and surface of endothelial and epithelial cells in the air-blood barrier. **Histograms of frequency distribution of cytoplasm volume density in endothelial (A) and epithelial (C) compartments and of total surface of the endothelial (B) and epithelial (D) compartments in control, after 3 and 5 hours of hypoxia and in cardiogenic edema. For simplicity of graphic presentation, volume density is presented as number of points falling in endothelial and epithelial compartments, while endothelial and epithelial surfaces are presented as number of intersections between the surface and the test lines.

Fig. 6A shows the frequency distribution of the volume of the endothelial cell compartment in the various groups. One can appreciate that in control (data pooled with sham) and after hypoxia exposure, the frequency distributions of cellular volumes depart from normality showing a marked skewness as the median value was smaller than the mean (Table 5 provides the results of the normality test). After 3h of hypoxia, the highest frequency distribution (67%) occurred for the smallest volume range and both the mean and the median values significantly decreased (Table 5). After 5 h of hypoxia, cell volume tended to return towards control values. By contrast, in the cardiogenic model group, endothelial volume significantly increased (both for the mean and the median values) as the distribution extended towards high cell volume values, remaining skew (Table 5). Fig. 6B shows the frequency histograms for total endothelial cell surface: the distribution appears fairly similar in control (pooled data with sham) and hypoxia while, in the cardiogenic edema group, the distribution of surface values extended towards higher values with a significant increase in mean and median values (Table 5).

**Table 5:** Statistics on frequency histograms of volume and surface distribution of endothelial compartment of the thin portion of the air-blood barrier. F and P indicate either failed or passed for the normality test. For the significance of the median values, the Dunn's method was used, while for that of the mean values the Holm-Sidak method was used.

	CONTROL		HYPOXIA 3 h		HYPOXIA 5 h		CARDIOG. EDEMA	
	Cell volume	Cell surface	Cell volume	Cell surface	Cell volume	Cell surface	Cell volume	Cell surface

Normality test	F	F	F	P	P	P	F	F
Skewness	1.53	1.36	1.24	0.77	0.67	0.98	1.07	1.39
Mean ± SE	6.5 ± 0.5	22.1 ± 0.3	4.6# ± 0.4	21.9 ± 0.3	5.9 ± 0.5	22.6 ± 0.4	16.2* ± 0.8	27.8* ± 0.7
Median	5.7	21	3.5 #	21	5.2	22	14*	25*

Mean ± SD. * significantly different (P < 0.05) relative to control and hypoxia exposure. # significantly different (P < 0.001) relative to control

A similar analysis was carried on the epithelial cells. The epithelial cell volume distributions (Fig. 6C) were substantially similar in control (pooled data with sham) and after 3 and 5 h of hypoxia exposure; in the cardiogenic edema group, the average volume significantly increased (Table 6) because of a shift in volume towards higher values, although the overall range of volume distribution was the same in all conditions. Fig. 6D reports the frequency histograms for the total surface of epithelial cells. Despite the mean surface values do not differ on comparing control (pooled data with sham) to 3 and 5 h of hypoxia exposure (Table 6), there was a considerable increase in frequency in the low range of surface values (a ten fold increase, from ~ 4 to ~ 40% for the surface range 16–20). In the cardiogenic edema group, the epithelial cell surface distribution was shifted towards higher values and indeed the mean and median surface values were significantly increased relative to the other groups (Table 6).

**Table 6:** Statistics on frequency histograms of volume and surface distribution of epithelial compartment of the thin portion of the air-blood barrier. F and P indicate either failed or passed for the normality test. For the significance of the median values the Dunn's method was used, while for that of the mean values the Holm-Sidak method was used.

	CONTROL		HYPOXIA 3 h		HYPOXIA 5 h		CARDIOG. EDEMA	
	Cell Volume	Cell Surface	Cell Volume	Cell Surface	Cell Volume	Cell Surface	Cell Volume	Cell Surface

Normality test	F	F	P	P	P	F	P	F
Skewness	1.53	2.9	0.48	0.1	0.99	1.52	0.46	1.64
Mean ± SE	6.8 ± 0.4	22.1 ± 0.4	6.2 ± 0.3	21.6 ± 0.3	7.5 ± 0.4	22.8 ± 0.4	11.5* ± 0.4	26.9* ± 0.5
Median	6	20	6	21	7	22	11*	25*

Mean ± SD. * significantly different (P < 0.05) relative to control and hypoxia exposure.

**Figure 7:**
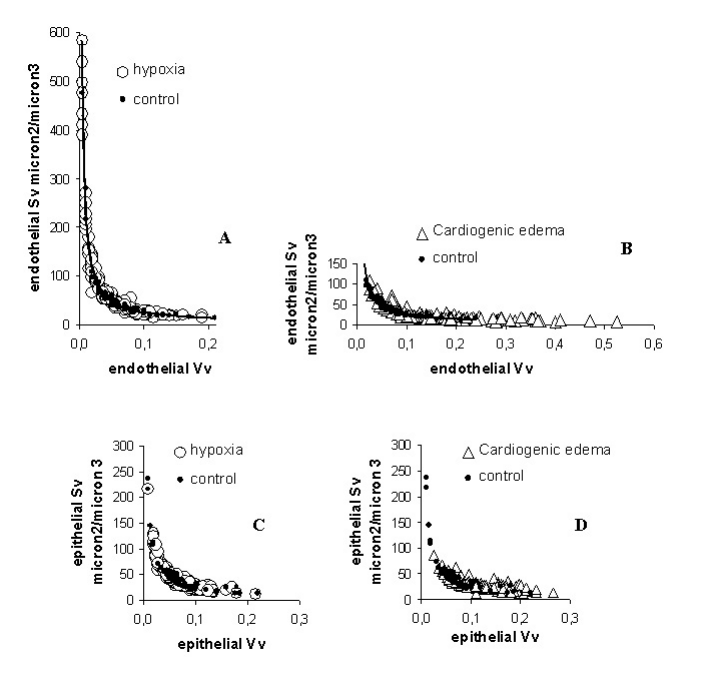
**Surface-volume relationships in endothelial and epithelial cells. **Surface and Volume densities (Sv and Vv, respectively) are presented for endothelial (A, B) and epithelial (C, D) cellular compartments. Panels A and C refer to control condition (closed circles) and hypoxia exposure (open circles); the data for 3 and 5 hours of hypoxia were grouped together. Panels B and D refer to control condition (closed circles) and to cardiogenic edema (open triangles). The continuous lines in panels A and B correspond to the iso-surface conditions.

Fig. 7 allows to better estimate the modifications induced on cellular morphology by either type of edema by plotting the plasma membrane surface to cell volume ratio (Sv) *vs *cell volume (Vv) for the endothelial and epithelial layers. These relationships are hyperbolic in nature and one can appreciate that in control conditions (closed circles, pooled data with sham) the data cover a wide spectrum of variation both in endothelial (Fig. 7A) and epithelial cells (Fig. 7C). In response to hypoxia (open circles, pooled data from 3 and 5 h), there is a definite trend for the data to scatter towards high Sv values and, correspondingly, very low cell volume in endothelial cells (Fig. 7A), while no significant variations, relative to control, were observed in epithelial cells (Fig. 7C). Conversely, in the cardiogenic edema model (open triangles), the data scatter towards high cell volume and correspondingly very low Sv values in endothelial cells (Fig. 7B), with no significant variations in epithelial cells (Fig. 7D).

**Figure 8:**
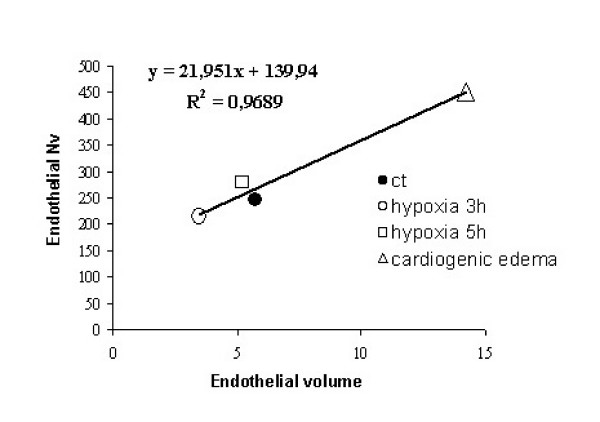
**Relationship between caveolar density and endothelial cell volume. **Regression between number of plasmalemmal vescicles per unit volume of endothelial cells (Nv) plotted vs the median values of endothelial cells volume in control (ct), after 3 and 5 h of hypoxia and cardiogenic edema.

Fig. 8 shows that a significant regression could be found by plotting caveolar density (Nv) in endothelial cell *vs *endothelial cell volume.

The W/D ratio was 4.98 ± 0.3 in control conditions (average data from zero time and sham for 3 h), 5.12 ± 0.1 and 5.16 ± 0.2 at 3 and 5 h of hypoxia exposure (for comparison, W/D was 5.42 ± 0.2 in the animals receiving saline infusion).

## Discussion

This study provides a contribution to the understanding of the cellular response to interstitial lung edema, a condition characterized by a relatively small increase in extravascular water, but considerable changes in interstitial space mechanics [2] and extracellular matrix composition [3]. particularly considering that recent findings led to hypothesize a specific "sensing" function by lung cells resulting from a perturbation in cell-matrix interaction [6].

### Plasma membrane composition

We found essentially no change in content of phospholipid phosphorous after 3 or 5 h of hypoxia. This finding is in line with the morphometric evaluation of lung cells in the air-blood barrier of animals exposed to similar hypoxic conditions indicating essentially minor changes in overall surface area of plasma membranes after hypoxia exposure, as also documented in isolated pulmonary endothelial cells [17]. This finding is at variance with the previous observation that phospholipid phosphorous was found to increase in response to saline infusion, reflecting in particular the increase in plasma membrane surface in endothelial cells [6,10].

Furthermore, the changes in pattern of total plasma membrane phospholipids differed in the two types of edema, as in hypoxia PC decreased and PE increased, while in saline induced edema they both increased [10].

PC is also an important component of surfactant phospholipids and therefore its modifications may potentially reflect changes in surfactant turnover. The increase in PE may contribute to the modification in lipid microenvironment surrounding plasma membrane channels whose activity could monitor changes in cell-matrix interaction [18,19].

We considered the plasmalogen production as this particular subset of phospholipids, by virtue of its ether bond, acts as endogenous antioxidant protecting cells and membrane from reactive oxygen species [20]. Plasmalogens were found to increase, as much as in the case of the saline induced edema [10]; this increase may buffer the decrease in glutathione level in hypoxia [21]. In the latter case we found no increase in lysophospholipids, suggesting no activation of PLA_2_, a finding similar to that observed in cultured endothelial cells exposed to 3% O_2 _for 4 h [17]. The no activation of PLA_2_, stems for a relative preservation of cellular integrity, considering that PLA_2 _activation in anoxia may lead to cellular death through a caspase independent mechanism by inducing nuclear shrinkage [22]. In line, with these findings, we did not find a significant increase of MDA, suggesting that the level of peroxidation remained unchanged in both models of interstitial edema.

### Membrane fluidity and cell surface-volume regulation

The biochemical determination of fluidity of plasma membrane is an estimate of its deformability that reflects its composition and the mobility of the lipid bilayer. Plasma membrane is relatively rigid as it can stretch elastically until the area increases up to 2–4%, beyond which rupture occurs [23]. Deformability increases when the phospholipids/cholesterol and PC/PE ratios increase. This occurred in the cardiogenic edema, allowing an increase in surface profile of endothelial cells [6], while opposite changes are found following hypoxia exposure.

Data of Fig. 6 and 7 also suggest that surface area and cell volume regulation are correlated and differently regulated in the two edema models, likely reflecting the specificity of cell-matrix mechanical interaction. Indeed, variations in cell volume may represent a step in the signalling process involving changes in the conductance of membrane ion channels [18,19].

### Lipid microdomains

Lipid microdomains include caveolae and lipid rafts that represent specialized sites of the plasma membranes since they host some important proteins implicated in signal-transduction [5,12]. We decided to monitor proteins that are known markers of either caveolae or lipid rafts. For caveolae we estimated the presence of cav-1, a structural protein responsible for the flask-like shape, and AQP1, a specialized protein channel found in endothelial cells for water and small nonionic molecules. In the present investigation we only determined the total amount of marker proteins, without determining their phosphorylated form, that is known to influence their redistribution and translocation from cytosol to membrane [24,25]. As specific marker of lipid rafts we considered CD55, a GPI-anchor proteins [26]. We also determined flotillin-1, a membrane protein expressed both in lipid rafts and caveolae [27,28]. After 3 h of hypoxia exposure, the decrease of cav-1 and AQP1 in DRF and their corresponding increase in IDFs and HDFs (Fig. 3), suggests an inhibition of the vesicle formation, as confirmed by the decrease in caveolar density in endothelial cells of the air-blood barrier (Fig. 8). These modifications are opposite to those previously documented in cardiogenic edema where caveolar expression was increased [5].

In the cardiogenic edema model, an increase in lipid microdomains was suggested by the increase in specific lipid components (PE, cholesterol). In hypoxia induced edema, where an inhibition of caveolae was found, the quantity of these lipids remained unchanged in DRF; therefore, this lends support to the hypothesis that these lipids may be used for lipid rafts formation. This hypothesis is strengthened by the observation that CD55 significantly increased at 3 h of hypoxia. In fact, each lipid raft can host a limited number of GPI- anchored protein [29], given the large size of its head, compared to the relatively small surface area of the lipid rafts. Therefore, an increased number of these molecules can only be accommodated by a corresponding increased number of lipid rafts. Since the total amount of flotillin-1 in DRF was unchanged, this further supports the hypothesis of a translocation of this protein from caveolae to lipid rafts. The inverse correlation between caveolin-1 and GPI-anchored protein was described in mutant cells lines either deficient in GPI biosynthesis or after overexpression of caveolin-1 [30].

A rearrangement of plasma membrane lipid microdomains suggests a modification in the expression of signal transduction proteins in response to the two edema models.

### Lung cellular response to interstitial edema

The morphometric studies indicate that mostly endothelial but not epithelial cells showed morphological changes in response to either type of edema, therefore, one may hypothesize that pulmonary interstitial edema (extracellular volume increase not exceeding 5%) evokes a response predominantly in endothelial cells as a result of a perturbation induced in their microenvironment. In both of our edema models, no alveolar flooding was present.

Alveolar epithelial cells regulate the volume and electrolyte composition of the alveolar lining fluid through the AQP5 and ENaC channels. For the level of hypoxia induced, no changes in AQP5 were found by western blotting (data not shown) and furthermore other data suggest that in mild hypoxia no inhibition of ENaC channels was found [31,32].

## Conclusion

We suggest a differential response of lung endothelial cells to cardiogenic and hypoxic pulmonary edema that reflect different pathophysiological mechanisms. In fact, the difference in the sequence of matrix macromolecules fragmentation [7] in the two models, might induce a specific cascade of cellular events from signalling – transduction to cellular functional attitude aimed at tissue repair.

## List of abbreviations

PGs: proteoglycans

PMC: plasma membrane fractions in control

PMH3 and PMH5: plasma membrane fractions at 3 and 5 h of hypoxia

DRF: Detergent Resistant Fraction

IDF: Intermediate Density Fraction

HDF: High Density Fraction

DBI: double bound index

MDA: malondialdehyde

DPH: 1, 6-diphenyl-1, 2, 5-hexatriene

PC: phosphatidylcholine

PE: phosphatidylethanolamine

PLA_2_: phospholipase A_2_

Vv: cellular volume density

Stot: total cellularsurface area

Sv: cellularsurface density (surface area per unit volume, μm^2^/μm^3^)

Sc: surface area of capillaries

N_v_: numerical density of plasmalemmal vesicles

PVs: plasmalemmal vesicles

W/D; fresh weight to dry weight tissue ratio

## Competing interests

The author(s) declare that they have no competing interests.

## Authors' contributions

LB and EB performed the biochemical studies; RD performed the morphometric studies; GM and PP conceived the study, participated in design and coordination and gave their contribution to write the manuscript.
